# Applications of virtual reality technology throughout the perioperative period in breast cancer patients: a scoping review

**DOI:** 10.3389/fonc.2026.1926450

**Published:** 2026-09-16

**Authors:** Yao Zheng, Qiaoqiao Yang, Yan Lan, Chunchang Zhong

**Affiliations:** 1 School of Nursing, Shanxi University of Chinese Medicine, Jinzhong, China; 2 Department of Breast Surgery, Women and Children’s Medical Center, Shenzhen Maternity and Child Health Hospital Affiliated to Southern Medical University, Shenzhen, Guangdong, China

**Keywords:** breast cancer, perioperative period, postoperative pain, preoperative anxiety, rehabilitation nursing, scoping review, virtual reality

## Abstract

**Objective:**

This scoping review aimed to summarize the application status, implementation methods, and effects of virtual reality (VR) technology in the perioperative period of breast cancer patients, so as to provide references for future research and clinical practice.

**Methods:**

In accordance with the Joanna Briggs Institute (JBI) scoping review guidelines, we systematically retrieved and analyzed relevant studies regarding VR technology applications in the perioperative management of breast cancer patients. Literature was searched from database inception to May 25, 2026, across nine Chinese and English databases, including the China National Knowledge Infrastructure (CNKI), Wanfang Data, VIP Chinese Periodical Database, Chinese Biomedical Literature Database (CBM), as well as PubMed, Web of Science, Embase, Cochrane Library, and CINAHL.

**Results:**

A total of 15 studies were included. VR technology was applied throughout the preoperative, intraoperative, and postoperative periods. Preoperative applications included immersive health education, operating room pre-adaptation, psychological adjustment, and surgical plan visualization. Intraoperative VR was used for attention diversion and pain relief. Postoperatively, VR mainly focused on upper limb rehabilitation training and optimizing patients’ rehabilitation experience.

**Conclusion:**

Existing studies have reported that VR interventions may alleviate perioperative anxiety and pain and facilitate functional rehabilitation among breast cancer patients. However, current VR intervention protocols are homogeneous and lack unified clinical intervention and evaluation standards. Further studies are needed to develop personalized nursing intervention protocols and establish standardized evaluation systems, so as to improve the clinical translation value of VR technology.

## 1 Introduction

Breast cancer is the most frequently diagnosed cancer in women worldwide. The 2024 GLOBOCAN projections, reported in 2026, estimated approximately 2.43 million incident cases of female breast cancer across the globe (1). Surgical resection serves as the cornerstone of curative treatment and long-term survival improvement for patients with early-stage breast cancer (2). Nevertheless, patients frequently suffer from a spectrum of perioperative physical and psychological morbidities, including postoperative pain, anxiety, and limb functional impairment. Left unmanaged, these adverse outcomes can substantially hinder postoperative rehabilitation, compromise patients’ quality of life, and reduce treatment compliance (3–6). As a non-invasive adjunctive intervention (7), VR technology has emerged as a promising strategy to optimize perioperative care for breast cancer patients. Notably, the clinical therapeutic benefits of VR interventions—such as enhanced limb mobility and upper extremity functional recovery—are typically fully manifested at approximately three months postoperatively. Accordingly, the present study focuses on research covering the time frame from 5 to 7 days preoperatively to 3 months postoperatively.

The application of VR technology in the perioperative care of breast cancer patients has expanded rapidly in recent years. Nevertheless, the current empirical evidence base remains fragmented. Existing review studies predominantly focus on single symptoms or discrete perioperative stages of breast cancer (3, 7), while few comprehensive analyses systematically summarize VR applications throughout the entire continuous perioperative workflow. Furthermore, there is a lack of standardized and systematic integration regarding VR intervention protocols, intervention frequency, outcome indicators, and therapeutic effects reported in primary studies.

A scoping review is well-suited to map existing evidence, characterize published studies, and identify research gaps in a targeted research field (8). On this basis, the present scoping review was conducted in accordance with the Joanna Briggs Institute (JBI) methodological framework for scoping reviews and the Preferred Reporting Items for Systematic reviews and Meta-Analyses extension for Scoping Reviews (PRISMA-ScR) reporting guidelines (9). This study aims to systematically elaborate on the application scenarios, intervention characteristics, outcome indicators, and clinical effects of VR technology in the perioperative period of breast cancer patients, thereby providing standardized references for future research design and clinical nursing practice.

## 2 Materials and methods

### 2.1 Research questions

This study was designed to address three core research questions as follows: (1) What are the application scenarios and intervention contents of VR technology in perioperative care for breast cancer patients? (2) What are the key characteristics of VR intervention protocols adopted for perioperative breast cancer patients? (3) What outcome indicators are commonly used to evaluate VR interventions, and what is the clinical effectiveness of such VR applications in the perioperative period of breast cancer surgery?

### 2.2 Inclusion and exclusion criteria

The inclusion and exclusion criteria were predefined prior to literature screening to ensure the rigor and consistency of study selection.

Inclusion criteria: (1) Study population: adult or adolescent patients diagnosed with breast cancer and undergoing perioperative management; (2) research theme: original studies focusing on the clinical application, implementation feasibility, intervention efficacy, or patient experience of VR technology; (3) research setting: perioperative care scenarios covering preoperative, intraoperative and postoperative stages of breast cancer surgery.

Exclusion criteria: (1) publications written in languages other than Chinese and English; (2) non-original studies, including systematic reviews, meta-analyses, and conference abstracts; (3) studies with unavailable full-text content or duplicate published data.

### 2.3 Search strategy

A comprehensive literature search was conducted across electronic databases from database inception to 25 May 2026.Chinese publications were identified from CNKI, Wanfang Data, VIP and CBM. The Chinese search terms included breast cancer, breast neoplasms, malignant breast tumors, virtual reality, VR technology, scenario simulation, virtual simulation, augmented reality, mixed reality, and perioperative period.

For English literature, searches were conducted in PubMed, Web of Science, Embase, CINAHL, and Cochrane Library. The English search formula was constructed with combined keywords: (“Breast Neoplasms” OR “Breast Cancer” OR “Breast Carcinoma” OR “Mammary Carcinoma”) AND (“Virtual Reality” OR “Augmented Reality” OR “Mixed Reality” OR VR OR “Immersive Virtual Reality” OR “Virtual Simulation” OR “Computer Simulation”) AND (perioperat* OR preoperat* OR intraoperat* OR postoperat* OR surg* OR operat*). All search terms were appropriately adjusted according to the retrieval rules of each database to maximize search coverage.

### 2.4 Literature screening and data extraction

All retrieved literatures were imported into EndNote 21 software for automatic and manual deduplication. Two independent researchers conducted rigorous two-round literature screening (primary screening by title and abstract, secondary screening by full-text review) and standardized data extraction. Any discrepancies regarding study inclusion were resolved by discussion or consultation with a third senior researcher to reach a consensus. The extracted data mainly contained basic study information (country/region, publication year, study design), research characteristics (sample size, intervention timing, intervention form, intervention frequency and cycle), and core research outcomes (primary outcome measures and evaluation tools).

### 2.5 Ethical consideration

This study is a secondary analysis of published literature. Therefore, ethical approval from an institutional review board was not required.

## 3 Results

### 3.1 Literature search results

The database search identified 641 records, and two additional records were retrieved through reference list screening, yielding a total of 643 records. Following duplicate removal, title and abstract screening, and full-text eligibility assessment, 15 studies (10–24) met the inclusion criteria and were included in the review. The study selection process is presented in **Figure 1**.

**Figure 1:**
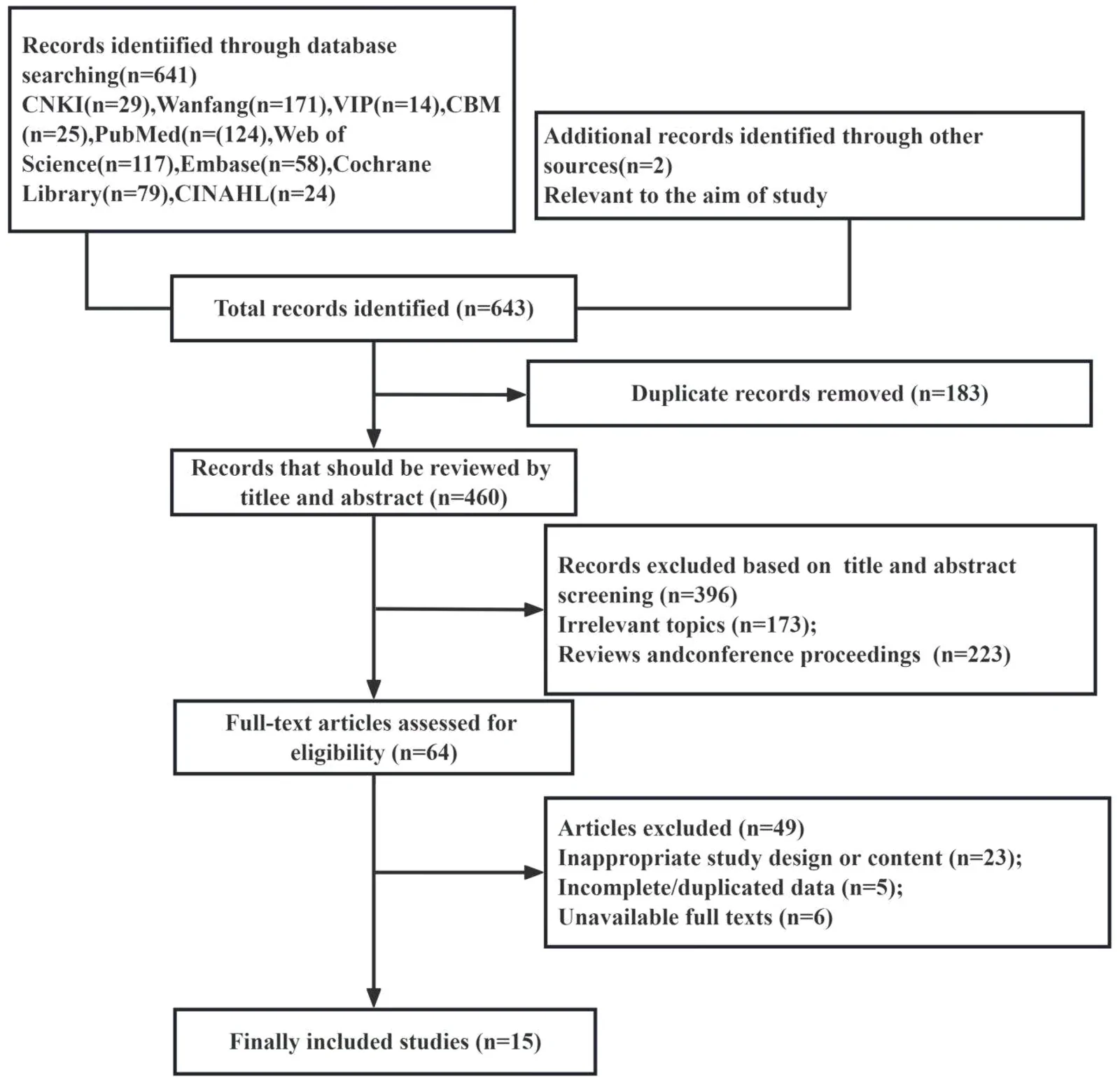
Flow diagram of literature screening.

The included studies were published between 2018 and 2026. Of these, six were conducted in mainland China (10, 11, 17, 19–21), three in Canada (13, 15, 16), two each in Poland (12, 14) and Turkey (18, 22), and one each in Egypt (23) and Taiwan, China (24). Detailed characteristics of the included studies are presented in **Table 1**.

**Table 1:** Basic characteristics of included studies (n=15).

Included studies	Country	Study type	Sample size	Timing of intervention	Intervention content		Intervention frequency and duration	Primary outcomes	Assessment tool
					Experimental group	Control group			
Zhang (10) et al. (2024)	China	RCT	52/52	Preoperative	Head-mounted VR equipment with 360° videos and voice guidance is adopted to conduct preoperative visits, anesthesia education and OR environment acquaintance.	Routine perioperative visit	Once	A, B	1, 2
Su (11) et al (2025)	China	RCT	40/40	Preoperative	VR goggles are used to play videos about surgery, anesthesia, postoperative pain relief and rehabilitation education.	Routine preoperative education	Once	A, B	1, 4
Oliver Czech (12)et al(2023)	Poland	RCT	9/7	Preoperative	VR virtual garden scenarios are applied for immersive relaxation and emotional regulation training	Routine nursing care	8 sessions, 15 min/session	A, E, F	6, 7, 12, 13
Najafi (13) et al(2024)	Canada	Descriptive study	166	Preoperative	Three-dimensional models implemented via AR enable dynamic visualization of various surgical approaches and corresponding postoperative cosmetic outcomes.	NO	Once	A, C	20
Czech (14) et al(2025)	Poland	Quasi-experiment	14/14/14/14	Preoperative	An immersive VR garden system was used to deliver meditation, breathing training and virtual environment exploration interventions	NO	5 sessions/week, 15–20 min/session, 2 weeks	A, E, F, G	5, 6, 7
Sommer (15)et al(2024)	Canada	Descriptive study	7/7	Preoperative	VR was used to simulate operating room scenarios, anesthesia induction and perioperative processes	Conventional nursing	12min	A, B	8, 9, 10, 11
El-Gabalawy (16)et al(2026)	Canada	RCT	12/11	Preoperative	Interactive VR simulation was performed to replicate the perioperative operating room setting and anesthesia induction procedure	Standard care	Once	A, B	8, 9, 10, 11
Liu (17) et al (2025)	China	RCT	47/44	Postoperative	VR goggles are worn during catheter placement for immersive panoramic videos of sea views, cultural scenery and cartoons	Conventional nursing	Continuous use during catheter placement	A, B, H, I	14, 15
Menekli (18)et al(2022)	Turkey	RCT	69/70	Postoperative	VR natural landscapes and gentle music were applied during catheter placement for patient relaxation	Conventional nursing	Continuous use during catheterization	A, B, H	2, 15
Jin (19) et al (2018)	China	RCT	60/60	Postoperative	The VR rehabilitation platform integrated with motion capture and virtual interaction is applied for upper limb function training	Conventional nursing	1 session/day, 30 min/session, 3 months	G	16
Zhu (20) et al (2019)	China	RCT	40/40	Postoperative	The constructed postoperative VR rehabilitation system delivers virtual task training to improve shoulder range of motion and facilitate lymphatic return	Conventional nursing	2 sessions/day, 15–30 min/session, 3 months	D, J	17
Jin (21) et al (2018)	China	RCT	38/38	Postoperative	Staged VR gamified rehabilitation intervention was implemented to improve joint mobility, prevent edema, and restore limb function	Conventional nursing	2 sessions/day, 15–30 min/session, 3 months	D, K	17
Feyzioğlu (22) et al(2020)	Turkey	RCT	20/20	Postoperative	With Xbox Kinect somatosensory sensing technology, VR virtual game tasks were adopted for shoulder functional training	Conventional nursing	2 sessions/week, 45 min/session, 6 weeks	B, G, J	15, 17
Yassa (23) et al(2024)	Egypt	RCT	30/30	Postoperative	VR-based motion feedback systems enable training of shoulder mobility and grasping function.	Pilates training	3 sessions/week, 40 min/session, 8 weeks	J, K	17, 18
Wu (24)et al(2024)	Taiwan, China	Phenomenological study	18	Postoperative	This study investigated patients’ VR rehabilitation experience as well as the benefits and drawbacks of VR application	NO	Early postoperative implementation	L	19

### 3.2 Application of VR technology in breast cancer patients during the perioperative period

#### 3.2.1 Preoperative application

Seven of the 15 included studies (10–16) investigated the preoperative application of VR technology in breast cancer patients. The primary intervention modalities reported in these studies were as follows: (1) immersive preoperative education (10), where VR systems were adopted to display information related to preoperative preparation, anesthesia processes and postoperative precautions; (2) operating room environmental familiarisation (10, 11, 15, 16), in which VR simulation was utilised to provide simulated exposure to the operating room environment, medical devices and anesthesia induction procedures; (3) psychological adaptation training (12, 14), with virtual natural or operating room environments deployed as an intervention strategy targeting emotional regulation and psychological desensitisation; and (4) surgical visualisation (13), in which VR was used to offer patients simulated previews of potential outcomes following breast reconstruction surgery.

#### 3.2.2 Intraoperative application

Two studies (17, 18) investigated the intraoperative application of VR technology. One study (17) implemented immersive VR scenarios, including seascapes, cultural landscapes, and animated cartoons, during peripherally inserted central catheter (PICC) placement. The other study (18) combined music with VR environments, such as parks, natural landscapes, and seaside walks, during venous port implantation procedures.

#### 3.2.3 Postoperative application

Six studies (19–24) investigated the postoperative application of VR technology in breast cancer patients. The main applications included: (1) technical support (21), in which motion-sensing devices captured joint movement trajectories and provided real-time visual feedback; (2) interactive rehabilitation design (19, 21, 22), involving immersive VR-based training environments integrated with gamified activities, such as virtual ball-throwing and fruit-cutting tasks; and (3) rehabilitation training, which initially focused on exercises involving the metacarpophalangeal and wrist joints during the early postoperative period (19–22, 24), followed by progressive resistance training and comprehensive upper-limb functional exercises as patients’ physical function improved (19–21, 23).

### 3.3 Characteristics of VR intervention protocols in the perioperative period of breast cancer patients

#### 3.3.1 Intervention equipment and technology

Immersive head-mounted displays were the primary VR devices used across the included studies (10, 12, 14, 15, 17, 18). Some studies also integrated motion-sensing interaction devices (16, 19, 20, 22) and motion capture systems (19, 20) to enable real-time movement feedback. The intervention content included 360° panoramic videos (10), virtual operating room environments (10, 11, 15), virtual healing gardens (12, 14), and gamified interactive rehabilitation scenarios (20, 22, 23).

#### 3.3.2 Intervention duration and frequency

The duration and frequency of VR interventions varied according to the perioperative stage. Preoperative interventions generally consisted of a single 10–30-minute session (10, 11, 13, 16), whereas intraoperative interventions were delivered throughout the entire PICC placement or venous port implantation procedure (17, 18). Postoperative rehabilitation programs typically lasted 6 weeks to 3 months, with sessions of 15–45 minutes delivered either once or twice daily (19–21) or two to three times weekly (22, 23). Follow-up periods ranged from immediate post-intervention assessment (17, 18) to 1–14 days (10–12, 14–16, 21–23) and up to 3 months after surgery (19, 20).

#### 3.3.3 Adherence to VR interventions

Among the included studies, participation rates ranged from 84.21% to 100%, completion rates from 81.32% to 100%, and dropout rates from 2.1% to 50% (10–24). Reported barriers to participation included VR-induced adverse effects and discomfort associated with wearing the equipment, particularly poor headset fit.

### 3.4 Outcome indicators and application effects of VR technology in breast cancer patients during the perioperative period

Across the included studies, a spectrum of clinical outcomes pertaining to perioperative VR interventions were documented, covering physiological function changes, psychological status, rehabilitation adherence, medical service experience, quality of life, and pain-related manifestations. Physiological indicators and physical function recovery: Data from eligible clinical trials suggest that preoperative and intraoperative VR interventions contribute to the stabilization of patients’ perioperative vital signs (11, 17, 18). Moreover, existing studies report that VR technology has the potential to shorten operative duration (17). Postoperative VR-integrated rehabilitation programs were recorded to correlate with improved shoulder range of motion, relieved upper-extremity lymphedema, and enhanced upper limb functional recovery in relevant study cohorts (20–23). Psychological and emotional outcomes: Cumulative findings from preoperative VR education and surgical simulation studies indicated reduced perioperative anxiety and depression among surgical populations (10–12, 14–16). One single-center study recorded lower postoperative decision regret regarding breast reconstruction in patients receiving three-dimensional virtual visualization guidance (13). For invasive procedures including PICC placement and venous port implantation, immersive VR distraction modalities were associated with mitigated procedural discomfort and alleviated negative emotional responses in clinical observations (17, 18). Rehabilitation adherence: Early postoperative VR-assisted rehabilitation protocols were documented to correspond with lower kinesiophobia and higher active participation in standardized rehabilitation exercises across relevant studies (10, 20–24). Healthcare experience and quality of life: Preoperative VR-based patient education was recorded to facilitate better intraoperative cooperation during anesthesia administration and elevate patients’ overall satisfaction with medical services (10, 11). Continuous perioperative VR intervention was also paralleled with improved postoperative sleep quality and optimized long-term quality of life in existing research datasets (12, 14, 19). Pain management: Trial data showed that preoperative VR intervention corresponded with milder postoperative pain intensity and reduced administration of rescue analgesics (10, 11). Intraoperative immersive VR distraction was observed to alter patients’ real-time pain perception during surgery (17, 18). Qualitative research findings further documented diminished subjective postoperative pain sensations among patients receiving postoperative VR immersion therapy (24).

## 4 Discussion

### 4.1 Phase-specific characteristics of VR applications during the breast cancer perioperative period

#### 4.1.1 Preoperative stage: cognitive education, psychological regulation, and surgical decision support

Existing studies have confirmed that preoperative anxiety, insufficient surgical cognition, and biased prognostic expectations may contribute to perioperative psychological stress and postoperative decision regret among patients with breast cancer (25, 26). Traditional preoperative education, including verbal instruction and printed pamphlets, is limited by insufficient communication time and individual differences in nurses’ expressive ability. In addition, static and abstract paper-based materials fail to provide patients with intuitive perceptual experience, resulting in limited efficacy in alleviating fear induced by unknown surgical scenarios (27, 28). Compared with conventional educational strategies, VR technology is capable of converting abstract surgical procedures and medical information into intuitive, interactive, and immersive visual scenarios (10–16). The current evidence synthesis indicates that preoperative VR intervention may effectively alleviate perioperative negative emotions and reduce the risk of postoperative decision regret in breast cancer patients (10, 11, 13, 15).

The underlying mechanisms may be explained from two aspects. On the one hand, immersive VR environments enable controlled preoperative scenario exposure, allowing patients to pre-experience the operating environment and relevant procedural stimuli. Such pre-exposure can reduce fear derived from subjective imagination and thereby relieve patients’ anxiety regarding surgery and anesthesia (29). On the other hand, AR visual projection technology can superimpose the postoperative somatic appearance corresponding to different surgical procedures on patients’ own bodies (13). Guided by mental simulation theory, this approach enriches patients’ concrete imagination of postoperative outcomes under different treatment options (30). Patients can intuitively compare cosmetic outcomes of various surgical schemes and select personalized procedures based on their own aesthetic preferences, which may reduce postoperative decision regret caused by one-sided cognitive anticipation.

Nevertheless, current preoperative VR interventions predominantly adopt a one-way passive viewing mode, in which patients merely receive virtual scenario information without independent control over experiential content and progress. Such single immersive observation has certain limitations in psychological adaptation and active cognitive regulation. With the continuous iteration of VR technology, bidirectional and interactive preoperative VR programs can be further developed to support independent operation and real-time interaction. Optimized VR intervention strategies are expected to improve preoperative cognitive education and psychological adjustment, while their clinical feasibility and practical value require further validation in future studies.

#### 4.1.2 Intraoperative stage: attentional diversion and acute pain and anxiety management

Current studies focusing on intraoperative VR-assisted interventions remain relatively scarce, with existing literature primarily concentrating on minimally invasive procedures under local anesthesia, such as peripherally inserted central catheter implantation and venous port insertion (17, 18). Patients remain fully conscious during these operations and are susceptible to multiple intraoperative stressors, including unfamiliar operating room environments, instrumental noise, and preoperative pain anticipation. These adverse factors commonly trigger tension, fear, and other negative affective states, while amplifying subjective pain perception (31, 32).

Traditional non-pharmacological strategies for relieving intraoperative pain and negative emotions mainly rely on verbal reassurance and background music intervention (33). However, such unidimensional interventions fail to sufficiently divert patients’ attention from traumatic procedural stimuli, resulting in limited analgesic and anxiolytic effects. In contrast, immersive VR intervention presents unique clinical advantages. The immersive virtual experience occupies patients’ multi-channel visual and auditory attentional resources and generates positive sensory stimulation, which may activate the mesolimbic reward pathway (34). Accumulated evidence from acute pain models has demonstrated that VR intervention can modulate cerebral metabolic patterns and suppress the activation of brain regions responsible for nociceptive processing and anxiety regulation (35).

Nevertheless, intraoperative VR application still has notable limitations. Wearing a VR head-mounted display during surgery restricts patients’ head movement, making it inapplicable to surgeries requiring constrained body positioning. Additionally, the placement of VR equipment and circuit layout may increase the difficulty of sterile management in the operating room. Furthermore, existing intraoperative VR studies are characterized by small sample sizes and single surgical scenarios, which are insufficient to support the generalized application of VR interventions across diverse surgical types, anesthesia modalities, and pain intensity levels. Further high-quality clinical studies are warranted to validate the clinical value of intraoperative VR intervention.

#### 4.1.3 Postoperative stage: functional rehabilitation promotion and quality of life improvement

Breast cancer patients often experience a series of postoperative adverse symptoms, including surgical incision pain, upper limb traction discomfort, and kinesiophobia (36). After discharge, the absence of ongoing professional guidance and rehabilitation supervision commonly results in poor rehabilitation adherence, which adversely impedes the recovery of upper limb functional performance (37). Traditional postoperative rehabilitation follow-up mainly relies on telephone calls and social media communication. Such single and monotonous intervention modes cannot provide real-time correction of movement errors, thereby failing to guarantee the standardization and sustainability of rehabilitation training (38).

As an alternative to conventional rehabilitation approaches, VR technology converts repetitive postoperative functional exercises into engaging interactive training through visualized functional demonstrations and gamified task design. This modality effectively boosts patients’ initiative and participation in rehabilitation. Equipped with instant visual feedback and positive motivational mechanisms (19, 20), VR-based training improves patients’ rehabilitation self-efficacy and enables standardized, consistent postoperative functional recovery practice (21, 22).

Nevertheless, the application of VR in home-based postoperative rehabilitation for breast cancer remains confronted with multiple limitations. The high cost of VR hardware restricts its popularization and accessibility in domestic rehabilitation scenarios. In addition, current studies predominantly focus on short-term rehabilitation outcomes, while long-term follow-up evidence is still lacking, leaving the sustained rehabilitation value of VR insufficiently validated (24). Future research directions include the development of low-cost home-use VR devices. Combined with tele-nursing and mobile health management strategies, a continuous and intelligent rehabilitation intervention system can be established to further refine and optimize the clinical application framework of VR technology in postoperative rehabilitation.

### 4.2 Limitations of current VR intervention protocols and outcome evaluation systems

#### 4.2.1 Small sample sizes and unstable intervention evidence

Among the 15 included studies, research regarding VR applications in breast cancer care has shown an increasing annual trend, with over 60% of the publications released after 2023 (10–17, 23, 24). However, most existing studies are small-scale exploratory investigations with insufficient statistical power, which carries a potential risk of overestimating the true intervention effect (39). In particular, small sample sizes are highly sensitive to participant dropout; even a small number of withdrawals can substantially alter the overall dropout rate, compromise data integrity, and limit the ability to objectively reflect the real-world clinical efficacy of VR intervention.

Several included studies reported a maximum dropout rate of up to 50%. The primary reasons for participant withdrawal are multifactorial. First, VR head-mounted displays may induce motion sickness symptoms, including dizziness, nausea, and visual fatigue, which reduce patient tolerance. Second, some patients are concerned that prolonged device wearing may interfere with surgical wound healing. Third, elderly patients often have poor adaptability to VR equipment operation. In addition, chemotherapy-related adverse reactions, transportation difficulties, and scheduling conflicts also contribute to voluntary participant withdrawal.

To reduce dropout risks in future clinical trials, preliminary VR adaptation experience is recommended before formal intervention. Optimizing headwear weight and single-session wearing duration, fully evaluating individual tolerance levels, and adopting flexible follow-up strategies can effectively improve participant retention and enhance the robustness of subsequent clinical evidence.

#### 4.2.2 Heterogeneity and non-standardization of outcome indicators and assessment tools

Currently, substantial heterogeneity and a lack of standardized assessment protocols exist in the outcome evaluation of VR interventions among breast cancer patients. Within the included studies, uniform assessment tools are absent for identical outcome indicators. For instance, different scales have been adopted to evaluate patient anxiety across studies, including the STAI. Accumulated evidence has confirmed that discrepancies in assessment tools may undermine the reliability and cross-study comparability of outcome results (40). Therefore, to accurately evaluate the genuine efficacy of VR interventions, future studies should prioritize standardized and universally recognized measurement instruments to reduce measurement bias derived from inconsistent assessment methods.

#### 4.2.3 Lack of patient stratification and individualized intervention design

Most existing studies regard breast cancer patients as a homogeneous population without conducting stratified analysis based on their clinicopathological characteristics (41, 42). Previous studies have demonstrated that patients undergoing different surgical procedures exhibit distinct degrees of physical function impairment, psychological stress levels, and rehabilitation demands, which may lead to differential responses to VR interventions (43). Nevertheless, current relevant studies have failed to perform such stratified investigations, limiting the capacity to guide individualized clinical nursing practice. Drawing on the paradigm of umbrella research (44), future studies can implement stratified management and personalized VR interventions according to patients’ clinical characteristics, so as to further improve the targeting efficiency and clinical application efficacy of VR-based rehabilitation strategies.

## 5 Conclusion

This scoping review synthesized data from 15 eligible studies to delineate the clinical application patterns, intervention features, and observed outcomes of VR technology within the perioperative care pathway for breast cancer patients. The results of the scoping review indicated that perioperative VR interventions are predominantly applied across three core clinical stages: preoperative psychological modulation, intraoperative analgesia and anxiolysis, and postoperative functional rehabilitation. Across the included literature, studies documented a range of favorable outcome signals associated with VR-assisted care, including reductions in perioperative anxiety and pain, improved upper limb functional recovery, and enhanced overall rehabilitation experience reported by participants. Notwithstanding these observed signals, the current evidence base is constrained by prevalent methodological limitations, including relatively small sample sizes, substantial inter-study heterogeneity in intervention protocols, and non-unified outcome assessment systems. To address these research gaps, future rigorous, multicenter, large-scale investigations are required to identify potential suitable target populations, develop standardized stage-specific VR intervention regimens, and build consistent evaluation benchmarks. The establishment of standardized VR intervention frameworks will further support evidence-based clinical translation and facilitate the standardized, large-scale implementation of VR technology in routine perioperative nursing and rehabilitation for breast cancer patients.

## Data Availability

The original contributions presented in the study are included in the article/**Supplementary Material**. Further inquiries can be directed to the corresponding author.
