# Supplementary material for: Applications of virtual reality technology throughout the perioperative period in breast cancer patients: a scoping review
Source: Front Oncol. 2026 Sep 16;16:1926450. doi: 10.3389/fonc.2026.1926450 (PMC13623584; doi:10.3389/fonc.2026.1926450)
Supplement: Supplementary file 1 [file DataSheet1.docx]

**PubMed search formula**

The complete search strategy developed for PubMed was as follows: ("Breast Neoplasms"[MeSH Terms] OR ("Breast Neoplasms"[Title/Abstract] OR "Breast Cancer"[Title/Abstract] OR "Breast Carcinoma"[Title/Abstract] OR "Mammary Carcinoma"[Title/Abstract])) AND ("Virtual Reality"[MeSH Terms] OR ("Virtual Reality"[Title/Abstract] OR "Augmented Reality"[Title/Abstract] OR "Mixed Reality"[Title/Abstract] OR VR [Title/Abstract] OR "Immersive Virtual Reality"[Title/Abstract] OR "Virtual Simulation"[Title/Abstract] OR "Computer Simulation"[Title/Abstract]) AND (perioperat*[Title/Abstract] OR preoperat*[Title/Abstract] OR intraoperat*[Title/Abstract] OR postoperat*[Title/Abstract] OR surg*[Title/Abstract] OR operat*[Title/Abstract]).
